# Urinary schistosomiasis: report of case diagnosed in bladder biopsy

**DOI:** 10.1186/s12907-018-0080-5

**Published:** 2018-11-28

**Authors:** Hafsa Chahdi, Amal Damiri, Mohamed Reda El Ochi, Mohamed Allaoui, Abderrahmane Al Bouzidi, Mohamed Oukabli

**Affiliations:** Department of Pathology, Military General Hospital Mohammed V, Mohammed V- Souissi University, Hay Riad, 10000 Rabat, Morocco

**Keywords:** Schistosomia haematobium, Hematuria, Bladder diseases

## Abstract

**Background:**

Urinary schistosomiasis is a common parasitic disease in endemic countries.

**Case presentation:**

We report the case of a patient who was on a working trip to Mauritania. This parasitosis, suspected in the presence of hematuria and the notion of stay in an endemic zone, was confirmed by the presence of Schistosoma heamatobium eggs during the histological examination of the bladder biopsy performed after cystoscopy, highlighting a bilharzial granuloma and of course, the diagnosis was confirmed by the presence of eggs during the direct examination of the freshly collected urine.

**Conclusions:**

It should be pointed out that the diagnosis of schistosomiasis must be evoked with the association of hematuria and the particular inflammatory aspect of the vesical mucosa and, of course, the notion of stay in an endemic zone.

## Background

Urinary schistosomiasis was discovered by Bilharz in Cairo and it is caused by the parasite *Schistosoma haematobium*. This endemic disease in 53 African countries, in the eastern Mediterranean and in India is suspected in the face of gross hematuria and confirmed by the detection of *S. haematobium* eggs. Cystoscopy, when performed, most often reveals diffuse bladder involvement that has been compared to “sugar grains” or “acne seeds” [1].

In Morocco, this pathology of importation is less well known. However, the diagnosis of bilharziasis must be mentioned and initial hematuria chart revealing a bladder tumor.

We report here an observation of a young patient who presented a pseudotumoral form of bladder schistosomiasis.

## Case presentation

A 25 year old man from Morocco worked in Mauritania as an engineer in a water dam for 1 year. One month after his return to Morocco, he has suffered abdominal pain and hematuria wrongly diagnosed in a local clinic as kidney stones.

**Fig. 1:**
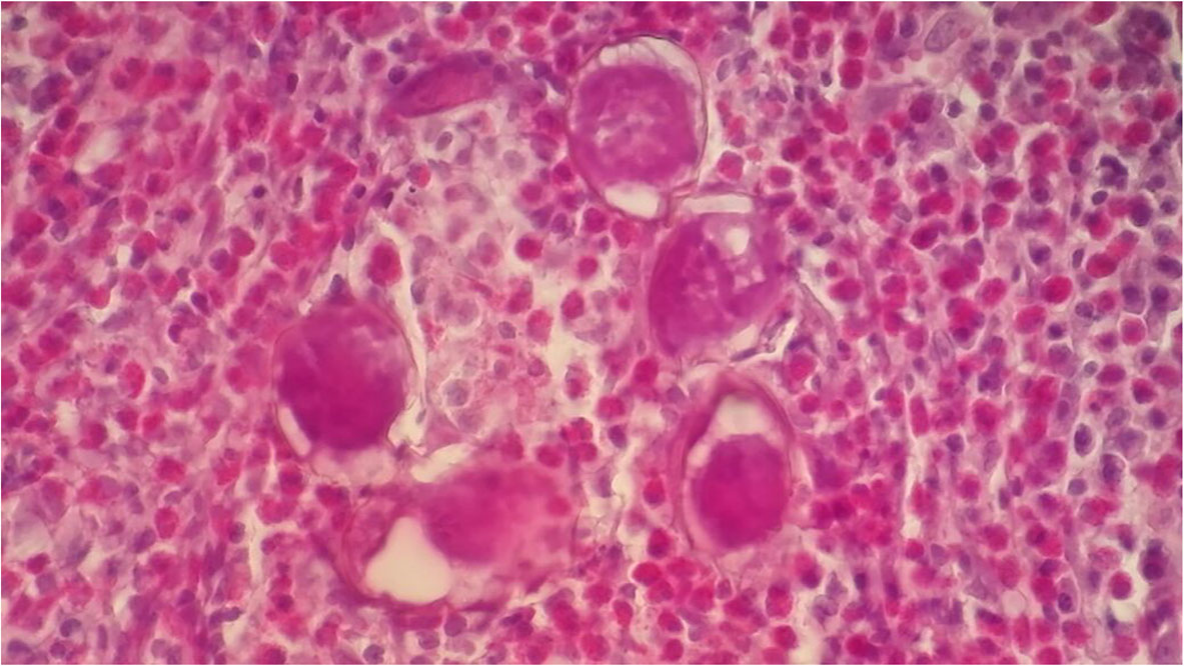
Histopathology of bladder mucosa shows the eggs of S haematobium surrounded by intense inflammatory infiltration in granuloma (hematoxylin and eosin stain, × 100)

He was admitted to a central hospital with progressive hematuria, he has benefited from a cystoscopy with biopsies. Histological examination of the biopsies revealed a granulomatous inflammatory reaction made of epithelioid and gigantocellular granulomas punctuated by eosinophilic polynuclear cells. These granulomas contain in their centers bilharzia eggs (Fig. 1).

**Fig. 2:**
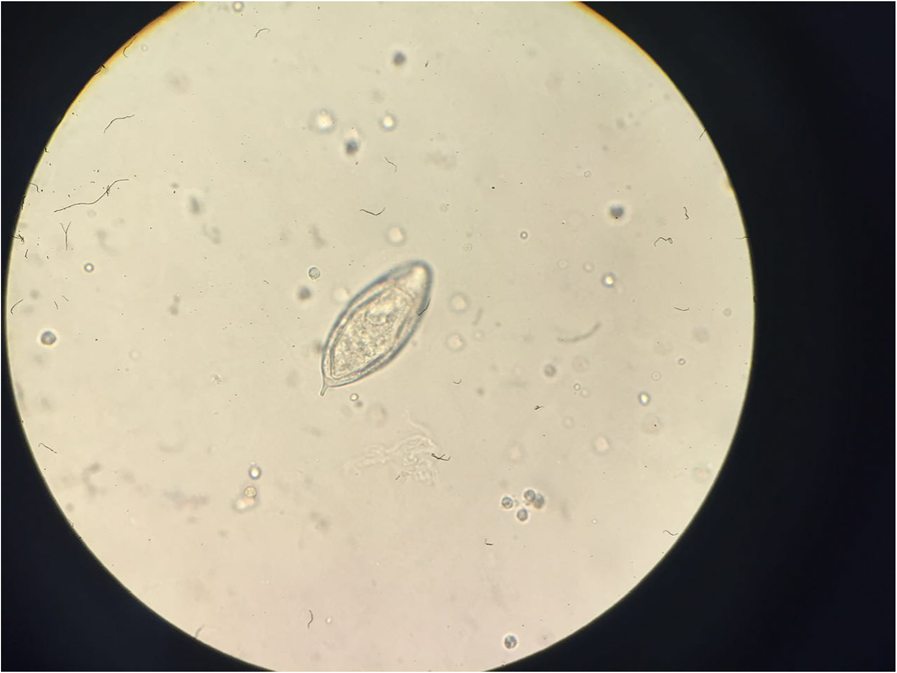
Sediment examination of a 24-h urine sample from case 1 demonstrates the diagnostic terminal spine of egg of Schistosoma haematobium (original magnification, × 400; no stain used)

The diagnosis was confirmed by the presence of *Schistosoma heamatobium* eggs in direct examination of fresh urine collected (Fig. 2).

## Discussion

Schistosoma is a subtype of trematodes, comprising multiple species. Of these, only five infect the human being, that is *Schistosoma mansoni*, *Schistosoma japonicum* [2–5], *S. haematobium*, *Schistosoma mekongi* and *Schistosoma intercalatum.* The first three are the most frequent. The only one that primarily infects the urinary tract is *S. haematobium*, causing urinary *schistosomiasis. S. haematobium* was discovered by a German physician, Theodor Bilharz, during an autopsy in Egypt in 1851 [5, 6]. To date, *S.haematobium* infection is still prevalent in sub-Saharan Africa and parts of the Middle East [2]. Our patient was in mauritania which is a contact point between north africa and sub-saharan africa. The life cycle of *S. haematobium* begins with the presence of eggs in the urine of affected patients; miracidia hatch and penetrate into intermediate hosts - snails; the development of cercariae and the release of snails into the water; the cercariae enter the human skin and migrate through the venous circulation into the liver, where the moat reaches maturity; adult flukes move to the venous plexus of the bladder, where female worms lay eggs; finally, the eggs migrate to the lining of the bladder and complete the life cycle [2, 7]. Our patient contracted the parasite through contact with contaminated water. The definite test for the diagnosis of urinary schistosomiasis is the identification of *S. haematobium* eggs in the urine.

Biopsy of an alleged bladder injury is another diagnostic tool. Eggs of *S. haematobium* have characteristic “terminal spines”, as has been demonstrated in our clinical case where a granulomatous inflammatory reaction has been observed around bilharzia eggs [8, 9]. The most important long-term complication of urinary schistosomiasis is the predisposition to bladder cancer [2]. The most common histological type of schistosomal bladder cancer is squamous cell carcinoma [8]. As a result, *S. haematobium* infection has been classified as a Group 1 carcinogen by the International Agency for Research on Cancer [5, 10].

## Conclusion

We reported a case of urinary schistosomiasis with typical histopathological features. Despite the limited prevalence of areas in sub-Saharan Africa and parts of the Middle East, the disease can still be seen in developed countries where the population is displaced.

## Abbreviation

S. haematobium: Schistosoma haematobium
